# Regulation of Tissue Regeneration by Immune Microenvironment–Fibroblast Interactions

**DOI:** 10.3390/ijms262411950

**Published:** 2025-12-11

**Authors:** Boram Son

**Affiliations:** Department of Bio & Fermentation Convergence Technology, Kookmin University, Seoul 02707, Republic of Korea; boramson@kookmin.ac.kr; Tel.: +82-2-910-6732

**Keywords:** tissue regeneration, fibroblast, immune microenvironment, extracellular matrix, inflammation, cell signaling

## Abstract

Tissue regeneration is a highly complex and dynamic process critically influenced by the immune microenvironment and its multifaceted interactions with fibroblasts. Traditionally regarded as structural cells responsible for extracellular matrix (ECM) production, fibroblasts have recently emerged as active regulators orchestrating immune responses and tissue repair. This review focuses on the reciprocal crosstalk between fibroblasts and key immune components, including macrophages, T cells, ECM, local pH, and signaling proteins. These interactions coordinate the initiation and resolution phases of inflammation, regulating fibroblast migration, proliferation, differentiation, and ECM deposition, which collectively determine the efficiency and quality of tissue repair. Special attention is given to the dynamic modulation of the immune microenvironment that governs fibroblast behavior during injury and regeneration. Finally, recent therapeutic strategies targeting this crosstalk—from molecular inhibitors to cell-based therapies—are discussed, highlighting emerging avenues for enhancing regenerative outcomes and mitigating fibrotic diseases. This integrated perspective positions fibroblast–immune interactions as a promising frontier in regenerative medicine, offering new opportunities for targeted tissue repair and control of chronic inflammation.

## 1. Introduction

Tissue regeneration is a highly orchestrated, multi-phase biological process essential for restoring the structural and functional integrity of organs and tissues after injury [1]. This complex process requires a well-balanced interplay of various cellular and molecular components within the local microenvironment, especially elements that regulate inflammation, extracellular matrix (ECM) remodeling, and resolution of tissue damage. Among these, the immune microenvironment—comprising diverse immune cells, ECM components, and soluble factors—has emerged as a crucial determinant of regeneration quality and outcomes [2].

Fibroblasts have historically been described as structural cells contributing to wound closure and scar formation via ECM deposition [3]. However, a growing body of evidence now recognizes fibroblasts as versatile and dynamic regulators actively orchestrating immune cell recruitment [4], cytokine production [5], and phenotypic modulation [6] throughout tissue repair. They exhibit remarkable plasticity, adapting to cues from surrounding immune cells (especially macrophages, T cells, dendritic cells) [7,8], soluble factors (interleukins, growth factors, chemokines) [9,10], and biophysical parameters such as pH and oxygen tension [11,12]. By integrating these signals, fibroblasts guide the progression from early inflammatory response through tissue remodeling to eventual restoration of homeostasis [13]. The fate and functional diversity of these cells can shape outcomes ranging from complete regeneration to chronic fibrosis [14].

The immune microenvironment itself is highly dynamic and context-dependent [15]. During acute injury, infiltrating immune cells initiate pro-inflammatory processes, releasing cytokines and reactive oxygen species (ROS) that activate local stromal populations including fibroblasts [8,16]. These signals not only promote fibroblast migration, proliferation, and differentiation but also regulate ECM composition through synthesis and degradation of collagen, glycoproteins, and matrix metalloproteinases (MMPs). Conversely, fibroblasts can secrete immunomodulatory factors that fine-tune leukocyte recruitment, polarization, and effector functions, establishing a reciprocal regulatory network [17]. Recent advances in single-cell RNA sequencing (scRNA-seq) and high-dimensional imaging have revealed distinct fibroblast subsets with specialized roles in immune regulation, tissue remodeling, and fibrosis across organs and diseases [18].

The physicochemical parameters of the tissue microenvironment, notably pH and oxygen levels, further influence fibroblast and immune cell function [19]. Local acidosis—commonly observed during inflammation and ischemia—modulates fibroblast phenotype, ECM interactions, and responsiveness to cytokines [20]. This fine balance of cell–cell, cell-ECM, and cell-soluble factor interactions is critical for successful tissue regeneration [21]. Dysregulation at any point can lead to ineffective repair, persistent inflammation, or excessive fibrosis.

Within this review, the reciprocal crosstalk between fibroblasts and the immune microenvironment—characterized by bidirectional communication where immune cells release cytokines such as tumor necrosis factor-α (TNF-α), interleukine-1β (IL-1β), and chemokines to activate fibroblast migration, proliferation, and myofibroblast differentiation, while fibroblasts in turn secrete immunomodulatory factors such as IL-10, C-X-C motif chemokine ligand 12 (CXCL12), prostaglandin E2 (PGE2) and ECM fragments that regulate immune cell recruitment, polarization, and effector functions—in tissue regeneration is systematically explored. Major discussion points include: (1) modulation of fibroblast function by immune cells, (2) ECM dynamics and their feedback on cellular behavior, (3) impacts of pH shifts and metabolic changes on repair processes, and (4) key signaling pathways enabling communication between fibroblasts and immune cells. In addition, the review outlines emerging therapeutic avenues that leverage these molecular and cellular interactions to promote effective tissue repair and control pathological fibrosis. Ultimately, a deeper mechanistic understanding of immune microenvironment-fibroblast interplay promises to advance regenerative medicine approaches and improve clinical outcomes in diverse tissue injury contexts.

## 2. Components and Features of the Immune Microenvironment

The tissue microenvironment is a highly integrated network composed of diverse chemical and cellular signals that collectively determine homeostasis and regenerative efficiency [22]. Especially in regeneration, the immune microenvironment—which includes immune cells, ECM, pH and metabolic cues, and molecular signals—plays a pivotal role in dictating fibroblast fate and tissue repair outcomes [23]. In Table 1, the key components and functional characteristics of the immune microenvironment involved in tissue regeneration are summarized.

### 2.1. Immune Cells

Immune cells rapidly infiltrate sites of tissue injury and play decisive roles in triggering inflammation and establishing the regenerative microenvironment [24]. Macrophages display remarkable plasticity—polarizing into pro-inflammatory M1 or anti-inflammatory M2 phenotypes—which dictates whether the outcome is regenerative or fibrotic [25]. M1 macrophages initially release inflammatory mediators to clear debris, followed by a timely M2 transition to promote fibroblast activation and ECM synthesis, essential for resolution and regeneration [26].

T cells also participate by modulating the regenerative and immunologic balance through interactions with stem cells and fibroblasts [27]. Further, dendritic cells (DCs), natural killer cells (NK cells), and other immune cell types function according to their specialized activities, contributing to inflammation resolution or progression [28]. Cytokines and growth factors released by immune cells are fundamental in shaping overall microenvironmental balance [29]. Direct reciprocal crosstalk between immune cells and fibroblasts is now recognized as a core axis governing the trajectory of tissue repair [30].

### 2.2. ECM

The ECM functions as more than a passive scaffold, serving as a dynamic platform for cell signaling and a reservoir and conveyor of growth factors [31]. Its principal components—collagen, fibronectin, elastin, and proteoglycans—impart organ- and context-specific properties to the local microenvironment [32]. The ECM modulates the concentration and spatial distribution of growth factors such as transforming growth factor-β (TGF-β) and fibroblast growth factor (FGF), critically influencing cell differentiation, migration, and survival [33].

During injury and regeneration, rapid ECM remodeling directs immune cell recruitment and activation while also shaping fibroblast migration, proliferation, and phenotypic modulation [34]. Enzymes such as MMPs regulate ECM stiffness and composition, thereby determining healing, regeneration, or fibrotic outcomes [35]. Furthermore, the ECM orchestrates immune cell adhesion, migration routes, growth factor docking, and mechanotransduction, serving as the central modulator of microenvironmental cues [36]. Disruptions in ECM dynamics are linked to impaired healing and pathological fibrosis [37].

### 2.3. pH and Metabolic Environment

Within the regenerative microenvironment, pH and metabolic state are key regulators of cellular activity, enzyme function, and signaling pathways [38]. Acute inflammation, hypoxia, and metabolic stress at injury sites cause local acidosis, which markedly influences both fibroblast and immune cell behavior [39].

Changes in pH alter growth factor and cytokine receptor structure and binding affinity, as well as modulate transcription factor activity. The enzymatic activities of MMPs and catalases are also pH-dependent, which in turn affects ECM remodeling, cell migration, and activation [40]. Acidic microenvironments regulate immune cell polarization and function, especially macrophage activation, and impact fibroblast migration and proliferation, collectively influencing inflammation resolution or scar formation [22]. Contemporary tissue engineering research emphasizes precise manipulation of local pH, oxygen, and metabolite concentrations to optimize regeneration.

### 2.4. Proteins and Signaling Molecules

Cytokines (e.g., TNF-α, IL-6, IL-10), growth factors (e.g., FGF, vascular endothelial growth factor; VEGF, epidermal growth factor; EGF), and chemokines (e.g., C-C motif chemokine ligand 2; CCL2, CXCL12) orchestrate intercellular communication and serve as master regulators at all stages of tissue regeneration [41]. These molecules modulate activation, differentiation, migration, and ECM remodeling of both immune cells and fibroblasts [42].

The local concentration and distribution of growth factors dictate cell survival and regenerative efficiency, while cytokines provide nuanced micro-regulatory signals critical for inflammation resolution [43]. Gene expression and signaling cascades are co-regulated by these proteins and molecular cues, directly shaping cellular fate.

Recent studies highlight the importance of these molecules in promoting angiogenesis, facilitating ECM remodeling, and supporting tissue repair, serving as the basis for novel therapeutic strategies including targeted signaling modulation and nanotechnology-based delivery systems.

**Table 1:** Key components and functional features of the immune microenvironment in tissue regeneration.

	Role and Function	Features and Impact	Refs
Immune Cells			
Macrophages	Exhibit plasticity polarized into pro-inflammatory M1 (debris clearance) and anti-inflammatory M2 (fibroblast activation, ECM synthesis) phenotypes	M1 promotes inflammation and pathogen clearance; M2 supports tissue repair and fibrosis resolution; balance determines regenerative or fibrotic outcomes	[24,25,26]
T Cells	Modulate regenerative and immunologic balance via interaction with stem cells and fibroblasts	Th1 cells promote inflammation; Th2 cells promote anti-inflammatory responses and fibrosis; regulating immune balance during repair	[27]
DCs	Antigen presentation and immune response modulation	Influence initiation and resolution of inflammation; key to immune system education	[28]
NK Cells	Cytotoxic activity and immune regulation	Play roles in eliminating infected or damaged cells; contribute to inflammation resolution	
ECM			
Collagen	Provides structural support and tissue strength	Major ECM component; influences cell adhesion, migration, and differentiation; altered composition affects healing and fibrosis	[31,32,33,34,35,36,37]
Fibronectin	Facilitates cell adhesion and migration	Acts as growth factor reservoir; mediates signal transduction pathways	
Elastin	Maintains tissue elasticity	Important for tissue resilience and function	
Proteoglycans	Modulate extracellular space, growth factor concentrations	Regulate water retention and bioavailability of signaling molecules	
MMPs	Mediate ECM degradation and remodeling	Critical for dynamic ECM turnover; regulate tissue repair vs. fibrosis depending on activity	[35]
pH and Metabolic Environment			
Local pH	Regulates receptor binding, enzyme activities, signal transduction	Acidic microenvironment alters cytokine signaling, immune cell polarization, and fibroblast functions	[22,38,39,40]
Oxygen Levels	Influences cellular metabolism and survival	Hypoxia triggers inflammatory and fibrotic pathways; affects angiogenesis	
Proteins and Signaling Molecules			
Cytokines			
TNF-α	Promotes inflammatory responses, activates immune cells, induces clearance of cellular debris at injury sites	Enhances NF-κB activity causing feedback loop formation, amplifies and sustains inflammation, high levels can cause tissue damage and toxicity	[41,42,43]
IL-6	Regulates diverse cell activation and differentiation, modulates acute inflammatory responses, involved in regeneration and fibrosis	Increases during infection and tissue damage, key to immune response regulation, chronic elevation promotes tissue damage	
IL-10	Anti-inflammatory cytokine, suppresses inflammation, maintains immune balance and protects tissues	Inhibits Th1 cytokines, promotes anti-inflammatory responses, reduces excessive inflammation and tissue damage	
Growth Factors			
FGF	Promotes cell proliferation, differentiation, migration, and angiogenesis	Central role in regeneration and wound healing, important for cell regeneration and ECM remodeling	[41,42,43]
VEGF	Stimulates angiogenesis, enhances oxygen and nutrient supply to damaged tissues	Essential for vascular regeneration and function recovery, improves oxygen and metabolic environment	
EGF	Stimulates epidermal cell proliferation and differentiation, supports wound healing and tissue regeneration	Directly contributes to cell proliferation and regeneration at wound sites	
Chemokines			
CCL2	Recruits monocytes and macrophages, promotes immune cell recruitment to inflammation sites	Essential for immune cell mobilization and activation in inflammatory and regenerative areas	[41,42,43]
CXCL12	Attracts stem cells and immune cells, promotes tissue regeneration and vascular remodeling	Key for creating regenerative environment, crucial for stem cell recruitment and function maintenance	

## 3. Biological Characteristics and Functions of Fibroblasts

Fibroblasts are the predominant stromal cells distributed throughout almost all connective tissues in the body, where they play essential roles in maintaining structural integrity, orchestrating wound repair, and directing tissue remodeling [14]. Characterized by an elongated spindle-shaped morphology and a prominent rough endoplasmic reticulum, fibroblasts possess remarkable plasticity that allows them to adapt to diverse tissue environments [44]. They remain in a quiescent state under homeostatic conditions but readily become activated in response to injury or stress signals, initiating cascades of cellular migration, proliferation, secretion, and matrix reorganization.

**Figure 1:**
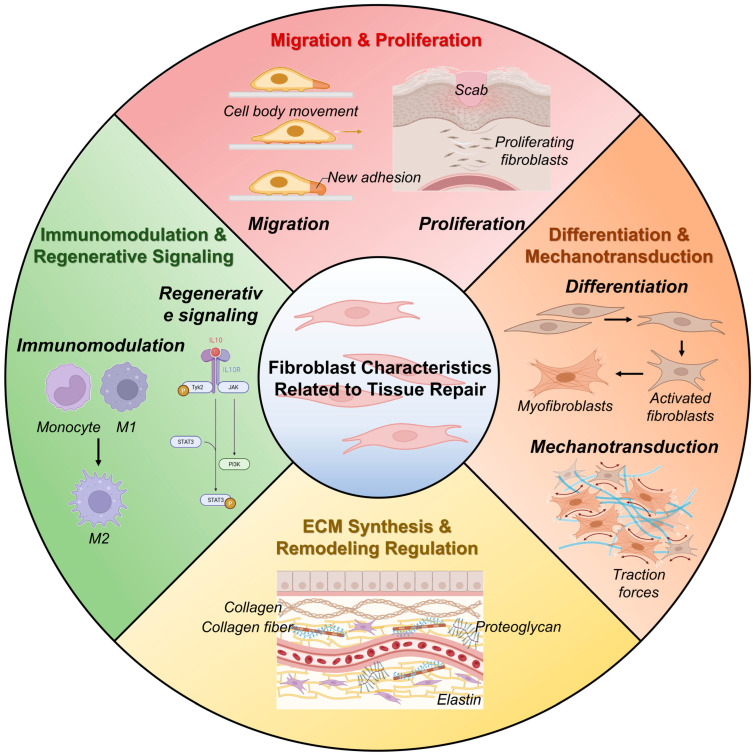
Overview of fibroblast characteristics and their pro-regenerative roles in tissue repair. Created in BioRender. Park, G. (2025) https://BioRender.com/8y89tg5 (accessed on 25 November 2025).

Beyond their structural capacity, fibroblasts actively integrate biochemical, mechanical, and immunological cues from their microenvironment [45]. By responding to growth factors, cytokines, and physicochemical changes such as pH and oxygen tension, fibroblasts serve as central coordinators of communication among immune cells, endothelial cells, and epithelial cells during tissue regeneration [8]. In Figure 1, their functions are broadly classified into four categories: migration and proliferation, differentiation and mechanotransduction [46], ECM synthesis and remodeling, and immunoregulation—all of which contribute in concert to the restoration of tissue integrity following damage.

### 3.1. Migration and Proliferation

Upon tissue injury, fibroblasts are among the first stromal cells to respond [8]. They are activated by inflammatory mediators and chemotactic cues, migrating toward the wound site where they proliferate to populate the damaged region. Key regulators of these processes include platelet-derived growth factor (PDGF), FGF, and TGF-β [47]. Activation of signaling cascades such as phosphatidylinositol 3-kinase (PI3K)/protein kinase B (Akt) and extracellular signal-regulated kinase (ERK)/mitogen-activated protein kinase (MAPK) promotes cytoskeletal remodeling and mitotic activity, enabling rapid coverage of the wound area [48].

During wound healing, fibroblast migration ensures the closure of tissue gaps and provides a supportive cellular scaffold for angiogenesis and re-epithelialization [49]. Their proliferative expansion replenishes stromal populations and sustains ECM production necessary for tensile strength. Dysregulation at this stage can delay healing or result in hypertrophic scarring, underscoring the balance between fibroblast activation and resolution [50].

### 3.2. Differentiation and Mechanotransduction

In the course of tissue repair, a subset of activated fibroblasts differentiates into myofibroblasts—cells endowed with α-smooth muscle actin (α-SMA)-rich stress fibers and contractile activity [51]. This differentiation is regulated by TGF-β/Smad signaling and influenced by mechanical tension generated within the local extracellular matrix. Through integrin-mediated mechanotransduction, fibroblasts sense ECM stiffness and convert these physical cues into biochemical signals that reinforce myofibroblast identity and promote wound contraction [52].

Mechanotransduction not only determines cellular phenotype but also dictates the mechanical integrity of the healing tissue [53]. Appropriate mechanosensing allows coordinated matrix deposition and compaction, ensuring functional restoration. In contrast, persistent mechanical stress or aberrant activation leads to myofibroblast persistence and excessive matrix accumulation, a hallmark of fibrotic disease [54]. Thus, regulating the fibroblast–matrix mechanosignaling axis is critical for achieving regenerative healing rather than pathological scarring.

### 3.3. ECM Synthesis and Remodeling Regulation

Fibroblasts are the principal producers and organizers of ECM components including collagens, elastin, fibronectin, and glycosaminoglycans [55]. These components establish a biochemical and mechanical niche that supports neighboring cells and maintains tissue architecture. The synthesis and organization of ECM molecules are dynamically adjusted during regeneration under the control of growth factors and matrix remodeling enzymes [56].

In wound healing, fibroblasts deposit provisional matrix components that serve as a scaffold for cell migration and vascular ingrowth [57]. As healing progresses, MMPs and their inhibitors (TIMPs) orchestrate ECM turnover, replacing temporary matrix with mature, tissue-specific structures [58]. Balanced ECM remodeling is vital: insufficient deposition weakens tissue repair, whereas excessive or dysregulated production can drive fibrosis. Therefore, fibroblast-mediated ECM homeostasis forms a cornerstone of effective regenerative outcome.

### 3.4. Immunomodulation and Regenerative Signaling

Beyond their canonical structural functions, fibroblasts play an essential role as anti-inflammatory and immunoregulatory cells that orchestrate the resolution phase of wound healing [59]. While they initially respond to pathogen- and damage-associated molecular patterns via receptors such as Toll-like receptors (TLRs), their subsequent activity shifts toward dampening excessive inflammation and promoting tissue repair [60]. Through the controlled release of cytokines and chemokines, including IL-10, CXCL12, and PGE2, fibroblasts modulate macrophage polarization from the pro-inflammatory M1 to the reparative M2 phenotype and support the recruitment of regulatory T cells [61]. These immunomodulatory functions enable fibroblasts to restore immune homeostasis, facilitate matrix remodeling, and prevent chronic inflammation that could otherwise lead to fibrosis.

Fibroblasts also modulate immune cell polarization: they can promote anti-inflammatory macrophage M2 phenotypes and regulatory T cell activity to facilitate repair, or conversely perpetuate chronic inflammation when persistently activated [7]. Through reciprocal paracrine communication, fibroblasts influence the tempo and quality of healing. Their ability to link immune regulation with matrix synthesis makes them indispensable to the timely resolution of injury and the prevention of maladaptive fibrosis [8].

## 4. Fibroblast Immune Regulation: Subsets, Organ Specificity, and Signaling

Recent advances in single-cell and spatial omics technologies have greatly deepened our understanding of the immune-regulatory roles and heterogeneity of fibroblast subsets [62]. Fibroblasts differentiate into various functional subtypes depending on tissue-specific characteristics and environmental contexts, with each subtype’s immune signaling network critically influencing tissue regeneration and pathological inflammatory responses. Thus, we comprehensively review the properties of immune-regulatory fibroblast subsets, their organ-specific functions, and the intercellular immune signaling pathways involved. Through this, we aim to elucidate the molecular and cellular mechanisms underlying fibroblast–immune system interactions during tissue regeneration.

### 4.1. Immune-Regulatory Fibroblast Subsets in Single-Cell and Spatial Omics

Utilizing the scRNA-seq and spatial omics technologies, remarkable heterogeneity of fibroblasts and their diverse immune-regulatory roles have been revealed across different tissues and disease contexts [62]. Therefore, fibroblasts have been redefined beyond their classical roles in ECM production and structural support to become multifaceted immune modulators, critically influencing immune cell infiltration, activation, and tissue remodeling. Notably, cancer-associated fibroblasts (CAFs) within tumor microenvironments exhibit distinct functional subtypes linked with patient prognosis and immune milieu modulation.

In lung cancer, single-cell profiling reveals three major CAF subsets: myofibroblasts, adventitial fibroblasts, and alveolar fibroblasts [63]. Myofibroblasts contribute to ECM stiffening and create an immunosuppressive niche associated with poor clinical outcomes, whereas adventitial and alveolar fibroblasts interact with immune cells to foster a more activated immune microenvironment tied to better prognosis. These subpopulations differ markedly in their ECM composition and secretory profiles, indicating specialized roles in lung tumor progression and immune regulation.

In skin cancer, CAFs segregate into matrix-producing CAFs (mCAFs) and immunomodulatory CAFs (iCAFs) [64]. The iCAF subset is characterized by high expression of proinflammatory cytokines and chemokines such as IL6, CXCL8, and MMP1, which modulate immune cell activity and facilitate tumor immune evasion. Importantly, iCAFs are enriched in highly invasive and malignant tumors, correlating with altered tumor-infiltrating T cell profiles and suppressed antitumor immune responses.

Further, hepatocellular carcinoma-associated fibroblasts have been demonstrated to engage immunoglobulin A (IgA), driving their conversion toward an immunosuppressive phenotype that attenuates CD8+ T cell-mediated antitumor immunity and enhances tumor immune evasion, revealing novel fibroblast-mediated immune checkpoint mechanisms [65].

Shifting focus to tissue regeneration, the immune-regulatory functions of fibroblast subsets have been elucidated in healthy skin and wound contexts [66]. Healthy skin harbors specialized fibroblast subsets such as CCL19^+^CD74^+^ antigen-presenting fibroblasts that maintain local immune niches. Upon injury or inflammation, IL11^+^MMP1^+^ inflammatory myofibroblasts emerge, secreting a variety of chemokines (e.g., CXCL5/6/8, CCL5/26, CXCL13) to recruit and activate neutrophils, monocytes/macrophages, and B cells, thus orchestrating immune cell infiltration and facilitating effective tissue repair and inflammation resolution.

In oral and cutaneous wound healing models, a distinct peroxiredoxin (Prx1)^+^ fibroblast subset expressing high levels of TLR2/4 activates nuclear factor κ-light-chain0enhancer of activated B cells (NF-κB) signaling to secrete key inflammatory chemokines like CXCL1 and CCL2. This chemokine production is crucial for early macrophage recruitment and microbial clearance at the wound site [67]. Disruption of this pathway results in impaired wound healing, persistent inflammation, and bacterial overgrowth, underscoring the indispensable role of these fibroblast subpopulations in immune-mediated tissue regeneration.

Collectively, these cutting-edge spatial and single-cell omics studies highlight fibroblasts as pivotal immune modulators shaping cytokine and chemokine networks, immune cell trafficking, and the balance between inflammation and resolution during tissue regeneration and disease. This integrated mechanistic insight offers promising avenues for targeted therapeutic strategies aiming to modulate fibroblast–immune crosstalk in regenerative medicine and oncology.

### 4.2. Tissue-Specific Fibroblast Subsets and Immune Signaling Pathways

Fibroblasts exhibit diverse differentiation and specialization patterns reflecting the unique microenvironment and physiological demands of each tissue. Organ-specific fibroblast subsets possess distinct molecular markers and unique cytokine and chemokine secretion profiles, contributing differently to immune cell infiltration, activation, and inflammation regulation.

In the kidney, fibroblasts act as key immune regulators in chronic kidney disease and fibrosis [68]. Single-cell transcriptomic analyses reveal kidney-specific fibroblasts secrete inflammation- and fibrosis-associated growth factors and cytokines, promoting immune cell infiltration and progression of tissue fibrosis [69]. Persistent activation of TGF-β signaling induces excessive ECM accumulation, maintaining a chronic inflammatory microenvironment through interactions with macrophages and T cells [70]. This distinct immunoregulatory feature provides crucial mechanistic insights for kidney regeneration and therapeutic strategies.

In cardiac tissue, specialized fibroblast subsets become activated upon injury, orchestrating inflammation and regeneration simultaneously [71]. Cardiac myofibroblasts modulate inflammatory responses at damaged sites by recruiting immune cells and secreting cytokines [72]. The production and regulation of anti-inflammatory cytokines, such as IL-10 and TGF-β, play a critical role in cardiac fibroblasts. Imbalance in these pathways is closely linked to chronic heart failure and fibrosis progression. Additionally, cardiac fibroblasts can reprogram the immune microenvironment by modulating macrophage polarization under hypoxic conditions [73].

Colonic mucosal fibroblasts are essential for innate immune responses and maintaining the epithelial barrier [74]. During inflammatory bowel disease (IBD), inflammatory fibroblast subsets upregulate secretion of chemokines like IL-6, CXCL1, and CCL2, promoting neutrophil and macrophage infiltration. These pro-inflammatory fibroblasts exacerbate chronic inflammation through NF-κB and signal transducer and activator of transcription 3 (STAT3) transcriptional pathways while other subsets contribute to mucosal healing by balancing immune responses.

These organ-specific immune regulatory features also arise due to differential expression of immune receptors like TLRs and major histocompatibility complex (MHC) molecules [75]. Certain fibroblast subsets activate NF-κB signaling via TLRs to regulate inflammation and interact directly with T cells through MHC class II expression, finely tuning immune responses [76]. Transcription factors STAT3 and NF-κB centrally mediate these signaling pathways, regulating fibroblast immunomodulatory activity.

Understanding these organ-specific immune regulatory mechanisms of fibroblasts is foundational for developing novel therapies that effectively modulate tissue regeneration and pathological inflammation.

## 5. The Role of Immune Microenvironment–Fibroblast Interactions

**Figure 2:**
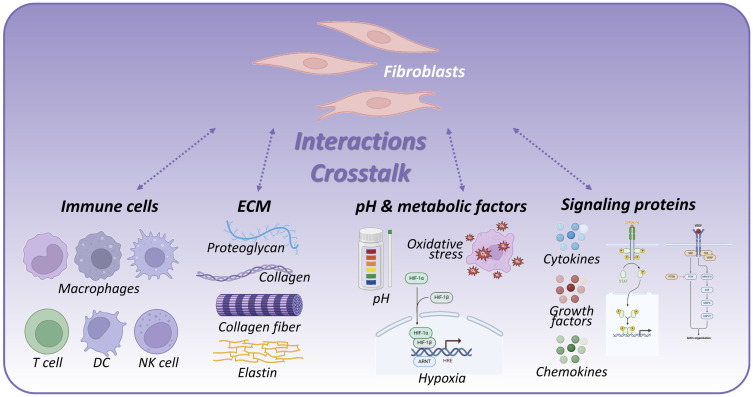
Fibroblast–immune microenvironment interactions regulating tissue regeneration. Created in BioRender. Park, G. (2025) https://BioRender.com/97xfa9y (accessed on 25 November 2025).

The interactions between fibroblasts and the immune microenvironment are pivotal in orchestrating the outcome of tissue regeneration, influencing the delicate balance between effective repair, chronic inflammation, or fibrosis. Figure 2 demonstrates the interactions between fibroblasts and immune microenvironment that regulate tissue regeneration, and Table 2 presents fibroblast crosstalk partners and their distinct functional contributions.

### 5.1. Fibroblast–Immune Cell Interaction Mechanisms

The interaction between fibroblasts and immune cells is mediated through several distinct pathways. Physical interactions allow direct contact between these cell types, enabling bidirectional communication that can alter cell activation status and influence the migration and positioning of both fibroblasts and immune cells within tissue compartments [7]. Molecular signaling is also fundamental to this crosstalk: fibroblasts secrete a variety of chemokines, growth factors, and ECM-derived fragments, each of which regulates immune cell recruitment, polarization, and survival, and in turn, immune cells release factors that modify fibroblast phenotype and function.

### 5.2. Impact of ECM Remodeling on Regeneration and Immune Cell Recruitment

Dynamic remodeling of the extracellular matrix by fibroblasts, mediated through the synthesis, deposition, and degradation of ECM components, has profound effects on the regenerative process [77]. Changes in matrix stiffness and composition alter the migratory routes for immune cells, regulate their adhesion and retention in damaged tissues, and shape local cell signaling cues [78]. Excessive or imbalanced ECM remodeling can create a pro-fibrotic environment, limiting regenerative efficiency and promoting chronic inflammatory cell infiltration, whereas controlled remodeling fosters effective tissue repair and the timely resolution of inflammation.

### 5.3. Physiological Impact of pH and Acidic Microenvironments

The metabolic landscape of the injury site induces fluctuations in pH, with local acidosis emerging as a key regulator of cell function [79]. Acidic conditions modulate gene expression in fibroblasts and immune cells, altering pathways such as MMP activity critical for ECM remodeling [80]. For example, decreased pH reduces MMP-1 and MMP-9 activities, resulting in impaired ECM turnover and excessive matrix accumulation, which fosters fibrosis progression. Additionally, local acidosis promotes fibroblast differentiation into myofibroblasts and enhances macrophage polarization toward the pro-fibrotic M2 phenotype, thereby perpetuating chronic inflammation and fibrotic responses.

### 5.4. Immune Cell-Derived Signals and Fibroblast Function

Immune cells, particularly macrophages and T cells, emit a repertoire of polarization and differentiation signals (such as cytokines and growth factors) that dictate fibroblast behavior [81]. M1-type macrophages promote a pro-inflammatory milieu that drives fibroblast activation and proliferation, while transition to the M2 phenotype—and the associated release of anti-inflammatory cytokines—facilitates fibroblast-mediated ECM synthesis, wound resolution, and attenuation of chronic inflammation [82]. T cell-derived factors similarly modulate fibroblast phenotype, ECM production, and even affect myofibroblast differentiation, ultimately dictating the efficiency of tissue regeneration and scar formation.

### 5.5. Role of Key Proteins (MMPs, Growth Factors, Chemokines)

Fibroblast–immune cell interactions are further refined by molecular mediators such as MMPs, various growth factors (FGF, TGF-β, VEGF), and chemokines (CCL2, CXCL12). Fluctuations in the local availability or activity of these proteins can either accelerate or impede tissue healing [54]. MMPs degrade ECM to facilitate cell migration and matrix remodeling, but overactivity can lead to tissue destruction or chronic wounds [83]. Growth factors determine fibroblast proliferation, migration, and their transition into myofibroblasts, while chemokines drive the recruitment and positioning of immune cells within the regenerating tissue. Imbalances in these signaling networks are often implicated in pathologic fibrosis or persistent non-healing wounds [84].

**Table 2:** Interaction partners of fibroblasts and their specific functional roles.

Fibroblast Interaction Partner	Specific Examples of Interaction	Refs
Immune Cells		
Macrophages	M1 macrophages promote fibroblast activation and ECM degradation; M2 macrophages secrete TGF-β to induce fibroblast proliferation and wound healing via AKT, ERK1/2, STAT3 pathways	[7]
T Cells	Fibroblasts interact with T cells via human leukocyte antigen class II (HLA-II), modulating helper T cell 1/2 (Th1/Th2) balance; Th2 cytokines (IL-4, IL-13) promote fibroblast transition to myofibroblasts enhancing fibrosis	
ECM	Fibroblast-deposited collagen increases ECM stiffness guiding macrophage migration; fibronectin fragments act as immune cell activators	[77,78]
pH and Metabolic Factors	Acidic microenvironment promotes fibroblast pro-fibrotic phenotype; low pH modulates MMP activity affecting ECM remodeling; lactate influences macrophage M2 polarization	[79,80]
Signaling Proteins		
Growth Factors	PDGF-D enhances cardiac fibroblast proliferation and migration; FGF and TGF-β regulate fibroblast–myofibroblast differentiation crucial for tissue repair	[54,83,84]
Chemokines	Fibroblast-produced CCL2 recruits monocytes/macrophages; CXCL12 attracts hematopoietic stem cells and lymphocytes facilitating regeneration and immune cell organization	

## 6. Therapeutic Strategies Targeting Fibroblast–Immune Microenvironment Interactions for Enhanced Tissue Regeneration

Building upon increasing knowledge of fibroblast biology and their multifaceted interactions within the immune microenvironment, recent research has emphasized that fibroblasts play a pivotal role in integrating immune signals, ECM remodeling, and local physicochemical conditions to orchestrate tissue repair. These reciprocal interactions not only determine the outcome of regeneration—balancing repair and fibrosis—but also provide a mechanistic foundation for therapeutic modulation of the regenerative niche [85]. Accordingly, emerging regenerative strategies are shifting from approaches that target individual cell types or isolated pathways toward therapies that precisely regulate fibroblast–immune microenvironment interactions [86,87]. By fine-tuning these cellular and molecular communications, it becomes possible to enhance constructive regeneration, promote inflammation resolution, and prevent pathological fibrosis [8,88].

### 6.1. Modulation of Fibroblast–Immune Crosstalk

Interventions that regulate the dynamic signaling exchange between fibroblasts and immune cells are emerging as powerful tools to enhance regeneration. Adjusting macrophage–fibroblast communication, for instance, can redirect macrophage polarization toward regenerative M2 phenotypes while simultaneously preventing fibroblast overactivation [89]. Similarly, modulating T cell–fibroblast dialog through selective cytokine or checkpoint pathways can establish a controlled inflammatory context that promotes resolution and matrix remodeling [90]. Targeted delivery of specific cytokines (e.g., IL-10, TGF-β modulators) or chemokine inhibitors (e.g., CCR2/CCL2 blockade) enables precise immunoregulatory manipulation without global immunosuppression [91].

### 6.2. Regulation of ECM–Fibroblast Signaling Network

Because fibroblasts both remodel and respond to ECM cues, therapeutic control of ECM composition and mechanics offers a route to influence immune–fibroblast coordination. Strategies include biomaterials that present immunomodulatory matrix ligands, decellularized scaffolds retaining native cytokine-binding motifs, or enzyme inhibitors that normalize matrix stiffness by regulating MMP and LOX activities [92]. Through restoring balanced ECM dynamics, fibroblasts can regain regenerative phenotypes and favor anti-fibrotic immune responses.

### 6.3. Targeting Physicochemical Microenvironmental Factors

Local biophysical and metabolic conditions—such as acidosis, hypoxia, and oxidative stress—profoundly shape fibroblast and immune cell interactions [93]. Innovative biomaterials such as pH-buffering hydrogels can neutralize acidic microenvironments, restoring MMP activities and ECM homeostasis [94,95]. Oxygen-releasing scaffolds alleviate hypoxia-induced pro-fibrotic signaling by normalizing cellular metabolism. Moreover, redox-modulating nanoparticles can decrease oxidative stress, attenuating fibroblast activation and limiting inflammation [96,97]. These strategies collectively create a favorable microenvironment for coordinated fibroblast–immune cell function [98]. For instance, poly lactic-co-glycolic acid (PLGA)-based oxygen carriers have demonstrated efficacy in resolving ischemia-related fibrosis in vivo [99].

### 6.4. Growth Factor and Chemokine Signaling–Mediated Regulation of Fibroblast Function

The activation state and differentiation trajectory of fibroblasts are primarily determined by the intensity and duration of signaling mediated by key factors such as TGF-β, FGF, PDGF, and the IL family [100]. TGF-β signaling modulation through selective inhibitors, such as SB431542 or neutralizing antibodies, prevents pathological fibroblast activation while permitting physiological ECM remodeling [49]. FGF and VEGF pathways can be simultaneously orchestrated using receptor tyrosine kinase agonists to enhance angiogenesis and tissue integration [101]. Chemokine axes, including CCL2-CCR2 and CXCL12-CXCR4, are targeted with small molecule antagonists to modulate immune cell recruitment and resolve chronic inflammation [102]. Advanced platforms, including nanoparticle-mediated delivery systems encapsulating siRNA against pro-fibrotic genes, and biomaterial-based controlled-release systems, enable spatiotemporal regulation of these signaling pathways, thereby improving tissue regeneration outcomes [103].

## 7. Conclusions and Future Perspectives

This review has presented an integrative perspective on the multifaceted roles of fibroblast–immune microenvironment interactions in tissue regeneration. By synthesizing existing evidence on ECM remodeling, immune cell-derived signaling, physicochemical factors such as local pH, and key molecular mediators, we have highlighted the reciprocal and dynamic nature of these cellular communications. The balance and precision of these cross-talks critically determine the efficiency of tissue repair, the resolution of inflammation, and the likelihood of fibrotic remodeling.

Despite remarkable scientific progress, several limitations and challenges remain for clinical translation. The heterogeneity, spatial diversity, and context-dependent phenotypes of fibroblast subpopulations complicate the prediction of therapeutic outcomes. Moreover, bidirectional communication between fibroblasts and both innate and adaptive immune cells is influenced by patient-specific factors such as underlying diseases, immune status, and genetic variability. Technical limitations persist in single-cell and spatial omics technologies, which are essential to delineate fibroblast subset functions and dynamic signaling within native tissue contexts.

In the clinical setting, particular attention must be paid to the safety and controllability of fibroblast-targeted or immune-modulating therapies. Unintended immune activation, off-target fibrotic responses, or aberrant tissue remodeling represent potential risks that require careful preclinical assessment. Issues such as biocompatibility of biomaterials, optimal dosing and delivery of cytokines or nanoparticles, and the potential for long-term genetic or epigenetic alterations in engineered cells must be systematically evaluated. Additionally, the regulatory landscape for cell- and gene-based regenerative therapies demands rigorous oversight to ensure reproducibility, ethical compliance, and patient safety.

Looking ahead, deeper mechanistic understanding of fibroblast–immune microenvironment crosstalk will accelerate the development of next-generation regenerative strategies. Integration of biomaterials engineering, targeted molecular therapies, nanotechnology-driven delivery, and advanced cell-based platforms—including genetically modified fibroblasts or mesenchymal stem cells—holds promise for overcoming current translational barriers. Rational and safe modulation of these interactions offers unprecedented opportunities to enhance repair, prevent fibrosis, and restore physiological tissue function after injury or disease. Continued multidisciplinary collaboration among immunologists, bioengineers, and clinicians will be essential to transform these conceptual advances into clinically effective and safe regenerative therapies.

## Data Availability

No new data were created or analyzed in this study. Data sharing is not applicable.
